# Supplementary material for: The effect of anchors and social information on behaviour
Source: PLoS One. 2020 Apr 14;15(4):e0231203. doi: 10.1371/journal.pone.0231203 (PMC7156041; doi:10.1371/journal.pone.0231203)
Supplement: S1 Appendix — (DOCX) [file pone.0231203.s001.docx]

**S1: Testing for order effects**

The first way one might consider testing for order effects would be to look at the effect of each possible order on donation decisions. However, since the orders of First Mover (FM) amounts presented to Second Movers (SMs) were randomized, the majority of amount orders only occur once. None occurred more than four times. Even if we only look at the order of the first two amounts shown, at most we have a sample size of 18 per unique order which is not sufficiently powered to assess order effects for each order. Hence, we proceed to test for order effects using a series of tests as set out below.

## Testing for influence of 2nd, 3rd, 4th, 5th, and 6th amounts shown.

In the main text, we present the results of testing for effects of the first amount shown on the donation decisions made by second movers. Below, we test if effects are similarly present for the 2nd, 3rd, 4th, 5th, and 6th amounts shown. To do this we dichotomized FM amounts (1=$0.50 and above, 0=less than $0.50) in the paper. As can be seen, we find no significant effects of the 2nd, 3rd, 4th, 5th, and 6th amounts shown oin the likelihood of SM transfers being greater than or equal to $0.50.

| **Comparing effects of first, second, third, etc. shown amounts** | | | | | | |
| --- | --- | --- | --- | --- | --- | --- |
|  |  |  |  |  |  |  |
|  | | | | | | |
|  | TRANSFER ORDER | | | | | |
|  | 1 | 2 | 3 | 4 | 5 | 6 |
|  | | | | | | |
| FM_amount1_binary | .619^**^ |  |  |  |  |  |
|  | (.234) |  |  |  |  |  |
|  |  |  |  |  |  |  |
| FM_amount2_binary |  | -.131 |  |  |  |  |
|  |  | (.237) |  |  |  |  |
|  |  |  |  |  |  |  |
| FM_amount3_binary |  |  | -.271 |  |  |  |
|  |  |  | (.237) |  |  |  |
|  |  |  |  |  |  |  |
| FM_amount4_binary |  |  |  | -.055 |  |  |
|  |  |  |  | (.237) |  |  |
|  |  |  |  |  |  |  |
| FM_amount5_binary |  |  |  |  | .129 |  |
|  |  |  |  |  | (.237) |  |
|  |  |  |  |  |  |  |
| FM_amount6_binary |  |  |  |  |  | -.293 |
|  |  |  |  |  |  | (.237) |
|  |  |  |  |  |  |  |
| Constant | 1.444^***^ | 1.835^***^ | 1.888^***^ | 1.794^***^ | 1.698^***^ | 1.905^***^ |
|  | (.169) | (.170) | (.159) | (.168) | (.173) | (.163) |
|  |  |  |  |  |  |  |
| N | 324 | 324 | 324 | 324 | 324 | 324 |
| R^2^ | .021 | .001 | .004 | 0.000 | .001 | .005 |
| Adjusted R^2^ | .018 | -.002 | .001 | -.003 | -.002 | .002 |
| Residual Std. Error (df = 322) | 2.108 | 2.129 | 2.126 | 2.130 | 2.129 | 2.125 |
| F Statistic (df = 1; 322) | 6.976^**^ | .307 | 1.299 | .054 | .296 | 1.536 |
|  | | | | | | |

Standard errors in parentheses, ^*^ *p* < 0.1, ^**^ *p* < 0.05, ^***^ *p* < 0.01.

**Testing for interaction of first and second amounts shown**

Another way to look for additional order effects is to examine a potential interaction between the first and second amounts shown. Again, as above we used dichotomized FM amounts. We find no evidence of an interaction between first and second amounts shown being greater than or equal to $0.50.

| **Testing for interaction of first and second amounts shown** | |
| --- | --- |
|  |  |
|  | |
|  | TRANSFER_SUM |
|  | |
| FM_amount1_binary | .510 |
|  | (.344) |
|  |  |
| FM_amount2_binary | -.153 |
|  | (.345) |
|  |  |
| FM_amount1_binary:FM_amount2_binary | .195 |
|  | (.475) |
|  |  |
| Constant | 1.534^***^ |
|  | (.264) |
|  |  |
| N | 324 |
| R^2^ | .022 |
| Adjusted R^2^ | .013 |
| Residual Std. Error | 2.114 (df = 320) |
| F Statistic | 2.384 (df = 3; 320) |
|  | |

Standard errors in parentheses, ^*^ *p* < 0.1, ^**^ *p* < 0.05, ^***^ *p* < 0.01.

**Testing for effects of order monotonicity**

One potential order effect was an effect of monotonicity. To test this, we coded each participant for if the order of FM amounts that they saw was monotonic increasing, monotonic decreasing, or not monotonic. We find no significant effects of order monotonicity on SM donation amounts.

| **Testing for effects of order monotonicity** | |
| --- | --- |
|  |  |
|  | |
|  | TRANSFER_SUM |
|  | |
| Monotonic_increasing | -.641 |
|  | (.394) |
|  |  |
| Monotonic_not | -.569 |
|  | (.311) |
|  |  |
| Constant | 2.242^***^ |
|  | (.274) |
|  |  |
| N | 324 |
| R^2^ | .012 |
| Adjusted R^2^ | .005 |
| Residual Std. Error | 2.121 (df = 321) |
| F Statistic | 1.879 (df = 2; 321) |
|  | |

Standard errors in parentheses, ^*^ *p* < 0.1, ^**^ *p* < 0.05, ^***^ *p* < 0.01.
