# Supplementary material for: The effect of anchors and social information on behaviour
Source: PLoS One. 2020 Apr 14;15(4):e0231203. doi: 10.1371/journal.pone.0231203 (PMC7156041; doi:10.1371/journal.pone.0231203)
Supplement: S2 Appendix — (DOCX) [file pone.0231203.s002.docx]

## S2: SM Responses to FM Contributions

## Distribution of SM Responses to Each Possible FM Contribution

The modal contribution is $0, accounting for 48.5% of all SM contributions. There is no significant difference between the proportion of zero contributions made in response to any of the FM transfers. The next most common transfer is $1, making up 11.4% of all SM contributions. transferring this amount.

In the following figure we summarise the above distributions in the form of a line graph depicting mean SM transfers in response to each FM transfer.

Aggregate SM Responses to FM Transfers. We do not present standard error bars in this figure as these are calculated on between-subject data, whilst the data presented here involves repeated observations per subject (i.e. within-subject’s data).
