# Supplementary material for: The effect of anchors and social information on behaviour
Source: PLoS One. 2020 Apr 14;15(4):e0231203. doi: 10.1371/journal.pone.0231203 (PMC7156041; doi:10.1371/journal.pone.0231203)
Supplement: S3 Appendix — (DOCX) [file pone.0231203.s003.docx]

## S3: Comparing mean SM transfers in response to different FM transfers

| **Hypotheses being tested** | **Paired pairwise 2-tailed t-test with Bonferroni correction (p-value)** | **Wilcoxon Signed Rank test with Bonferroni correction (p-value)** |
| --- | --- | --- |
| SM response to $0 = SM response to $0.10 | 1.000 | 1.000 |
| SM response to $0 = SM response to $0.25 | 1.000 | 1.000 |
| SM response to $0 = SM response to $0.50 | 1.000 | 1.000 |
| SM response to $0 = SM response to $0.75 | 1.000 | 1.000 |
| SM response to $0 = SM response to $1 | 0.045** | 0.035** |
| SM response to $0.10 = SM response to $0.25 | 1.000 | 0.072* |
| SM response to $0.10 = SM response to $0.50 | 0.150 | 0.043** |
| SM response to $0.10 = SM response to $0.75 | 0.035** | 0.097* |
| SM response to $0.10 = SM response to $1 | 0.000*** | 0.012** |
| SM response to $0.25 = SM response to $0.50 | 0.298 | 1.000 |
| SM response to $0.25 = SM response to $0.75 | 0.141 | 0.594 |
| SM response to $0.25 = SM response to $1 | 0.002*** | 0.021** |
| SM response to $0.50 = SM response to $0.75 | 1.000 | 1.000 |
| SM response to $0.50 = SM response to $1 | 0.006*** | 0.018** |
| SM response to $0.75 = SM response to $1 | 0.141 | 0.003*** |
