# Supplementary material for: The effect of anchors and social information on behaviour
Source: PLoS One. 2020 Apr 14;15(4):e0231203. doi: 10.1371/journal.pone.0231203 (PMC7156041; doi:10.1371/journal.pone.0231203)
Supplement: S4 Appendix — (DOCX) [file pone.0231203.s004.docx]

## S4: SM Responses to the Initial Amount (the anchor)

The figure below shows how SM’s responded to the *initial stated contribution* provided by each FM, i.e. the contribution stated in response to the initial amount (IA) presented to them. This provides an indication of the initial impact of different social information values. Thus, the results presented in 0 entail one observation per SM. A one-way ANOVA test shows a statistically significant difference between mean contributions in response to each anchor (F(5,318)=2.99, p=0.0117). Additional parametric and non-parametric pairwise tests comparing SM contributions to IAs can be found in the table below.

SM Response to Initial Amount Presented in Strategy Experiment

As can be observed, the initial contribution in response to the IA is less ‘temperate’ than the vector of contributions provided in response to all possible six FM contributions (Figure 1 in main text). Compared to the modest slope observed in Figure 1, there is a sharp discontinuity between responses to IA <$0.5 and responses to IA≥$.50, with the increase occurring somewhere in the range $0.25<IA≤$0.50. We also note no significant variation in SM responses *within* the intervals $0<IA<$0.5, and $0.50≤IA≤$1. The difference in the pattern of responses to the IA versus the full strategy data may reflect different thinking processes; for example, emotions might play a larger role in determining responses to the first amount seen (Brandts & Charness, 2011) compared to subsequent amounts. We cannot however confirm whether this is the case, as we did not elicit emotions-based data.

Comparing SM transfers in response to initial amount presented (*one observation per SM*)

| **Hypotheses being tested** | **T-test (2-tailed)**  **(p-value)** | **Mann-Whitney**  **(p-value)** |
| --- | --- | --- |
| SM response to $0 = SM response to $0.10 | 0.2856 | 0.8791 |
| SM response to $0 = SM response to $0.25 | 0.3460 | 0.8847 |
| SM response to $0 = SM response to $0.50 | 0.1406 | 0.0643* |
| SM response to $0 = SM response to $0.75 | 0.1565 | 0.0617* |
| SM response to $0 = SM response to $1 | 0.2129 | 0.0719* |
| SM response to $0.10 = SM response to $0.25 | 0.9386 | 0.4611 |
| SM response to $0.10 = SM response to $0.50 | 0.0174** | 0.0233** |
| SM response to $0.10 = SM response to $0.75 | 0.0187** | 0.0211** |
| SM response to $0.10 = SM response to $1 | 0.0278** | 0.0239** |
| SM response to $0.25 = SM response to $0.50 | 0.0051*** | 0.0192** |
| SM response to $0.25 = SM response to $0.75 | 0.0057*** | 0.0197** |
| SM response to $0.25 = SM response to $1 | 0.0098*** | 0.0244** |
| SM response to $0.50 = SM response to $0.75 | 0.8754 | 0.8952 |
| SM response to $0.50 = SM response to $1 | 0.7749 | 0.8305 |
| SM response to $0.75 = SM response to $1 | 0.8876 | 0.9307 |
