# Supplementary material for: The effect of anchors and social information on behaviour
Source: PLoS One. 2020 Apr 14;15(4):e0231203. doi: 10.1371/journal.pone.0231203 (PMC7156041; doi:10.1371/journal.pone.0231203)
Supplement: S5 Appendix — The dependent variable is cents transferred per second mover to the recipients. (DOCX) [file pone.0231203.s005.docx]

S5: Regressions on Second-Mover Transfers, using only SMs in groups without SM dropouts (n=279 SMs). The dependent variable is cents transferred per second mover to the recipients.

|  | **(1)** | **(2)** | **(3)** | **(4)** |
| --- | --- | --- | --- | --- |
|  |  |  |  |  |
| IA=$0 | -9.357 |  | -13.044* |  |
|  | (7.172) |  | (7.481) |  |
| IA=$0.10 | -16.212** |  | -17.889** |  |
|  | (7.8350) |  | (7.817) |  |
| IA=$0.25 | -11.079 |  | -14.388* |  |
|  | (6.974) |  | (7.346) |  |
| IA=$0.75 | 3.616 |  | -0.124 |  |
|  | (7.371) |  | (7.681) |  |
| IA=$1 | -4.626 |  | -9.514 |  |
|  | (7.353) |  | (7.696) |  |
| IA dichotomous (where 1≥$0.50, 0<$0.50) |  | 11.473*** |  | 11.619*** |
|  |  | (4.062) |  | (4.243) |
| Order in which FM transfer presented | -0.309 | -0.309 | -0.292 | -0.317 |
|  | (0.211) | (0.211) | (0.205) | (0.204) |
| FM transfer (cents) | 0.047*** | 0.047*** | -0.022 | 0.049** |
|  | (0.016) | (0.016) | (0.040) | (0.022) |
| Female | 11.524*** | 11.532*** | 11.524*** | 11.532*** |
|  | (4.215) | (4.236) | (4.215) | (4.236) |
| Age | 0.440** | 0.427** | 0.440** | 0.427** |
|  | (0.175) | (0.176) | (0.175) | (0.176) |
| Income (divided by 1000) | -0.068 | -0.078 | -0.068 | -0.078 |
|  | (0.057) | (0.058) | (0.057) | (0.058) |
| *Interactions* |  |  |  |  |
| IA=$0*FM transfer |  |  | 0.085 |  |
|  |  |  | (0.055) |  |
| IA=$0.10*FM transfer |  |  | 0.039 |  |
|  |  |  | (0.055) |  |
| IA=$0.25*FM transfer |  |  | 0.076 |  |
|  |  |  | (0.054) |  |
| IA=$0.75*FM transfer |  |  | 0.086 |  |
|  |  |  | (0.056) |  |
| IA=$1*FM transfer |  |  | 0.113** |  |
|  |  |  | (0.055) |  |
| IA dichotomous*FM transfer |  |  |  | -0.003 |
|  |  |  |  | (0.032) |
| Constant | 17.712** | 7.077 | 20.642** | 7.027 |
|  | (8.962) | (7.952) | (9.13) | (8.018) |
| Number of observations | 1638 | 1638 | 1638 | 1638 |
| Number of groups (i.e. SMs) |  |  |  |  |
| Wald Chi2 | 49.45*** | 46.33*** | 55.82*** | 46.53*** |
| Likelihood ratio test: mixed versus linear model | 2072.27*** | 2081.88*** | 2083.97*** | 2081.9*** |

Standard errors in parentheses, * p<0.1, ** p<0.05, *** p<0.01
