# Supplementary material for: The effect of anchors and social information on behaviour
Source: PLoS One. 2020 Apr 14;15(4):e0231203. doi: 10.1371/journal.pone.0231203 (PMC7156041; doi:10.1371/journal.pone.0231203)
Supplement: S6 Appendix — (DOCX) [file pone.0231203.s006.docx]

## S6: SM Responses to FM contributions disaggregated by IA (dichotomous)
