# Supplementary material for: The effect of anchors and social information on behaviour
Source: PLoS One. 2020 Apr 14;15(4):e0231203. doi: 10.1371/journal.pone.0231203 (PMC7156041; doi:10.1371/journal.pone.0231203)
Supplement: S7 Appendix — Individuals dummies for each anchor (reference category: self-interested). (DOCX) [file pone.0231203.s007.docx]

## S7: Multinomial Logit Model of Determinants of SM Type. Individuals dummies for each anchor (reference category: self-interested)

|  | **Conformist** | **Compensator** | **Unconditional** | **Other** |
| --- | --- | --- | --- | --- |
|  |  |  |  |  |
| IA=$0 | -0.592 | -2.601** | -0.615 | -0.083 |
|  | (0.670) | (1.111) | (0.514) | (0.621) |
| IA=$0.10 | -0.333 | -1.710** | -1.102* | -1.364 |
|  | (0.676) | (0.862) | (0.602) | (0.884) |
| IA=$0.25 | -0.427 | -15.629 | -0.435 | -0.146 |
|  | (0.650) | (67.235) | (0.503) | (0.634) |
| IA=$0.75 | 0.178 | -1.869** | 0.002 | -0.876 |
|  | (0.610) | (0.857) | (0.483) | (0.724) |
| IA=$1 | 0.327 | -1.527* | -0.399 | 0.364 |
|  | (0.627) | (0.874) | (0.546) | (0.641) |
| Income (div by 1000) | 0.005 | 0.001 | -0.007 | -0.006 |
|  | (0.005) | (0.008) | (0.005) | (0.006) |
| Age | 0.014 | -0.022 | 0.025* | -0.018 |
|  | (0.016) | (0.032) | (0.014) | (0.020) |
| Female | 0.905** | 0.719 | 0.820*** | 0.537 |
|  | (0.355) | (0.578) | (0.301) | (0.386) |
| Constant | -2.058*** | -0.340 | -1.024* | -0.344 |
|  | (0.765) | (1.126) | (0.615) | (0.812) |
|  |  |  |  |  |
| Chi2 | 57.901*** |  |  |  |
| n^a^ | 311 |  |  |  |

Standard errors in parentheses; * p < 0.1, ** p < 0.05, *** p < 0.01

^a^ Missing data from 10 respondents on income, age and gender (refusal to answer)
