# Supplementary material for: The effect of anchors and social information on behaviour
Source: PLoS One. 2020 Apr 14;15(4):e0231203. doi: 10.1371/journal.pone.0231203 (PMC7156041; doi:10.1371/journal.pone.0231203)
Supplement: S8 Appendix — (DOCX) [file pone.0231203.s008.docx]

## S8: Analysing Open-Ended Explanations for Transfer Decision

The robustness of the classification scheme can be informally assessed by analysing responses to an open-ended follow-up question that asked participants “*How did you decide on the amount that you contributed?”* Almost every player (n=304) answered this question. Reasons were coded manually using a coding frame that was designed to identify a) whether the SM explained their decision with reference to other players (FM, other SMs, or recipients), b) whether they explain their decision in terms of other motivations such as ‘greed’, ‘fairness’ or empathy, and c) whether they explain their strategy (conformism versus compensation versus other). Coding was conducted independently by three individuals initially; there was a medium level of interrater reliability (agreement for 82% of all items across all three researchers; Gwet’s AC (agreement coefficient) = 56%) and final codes were agreed on through a process of discussion. Results of the classification are presented below.

Results (see Figure below) suggest a high level of agreement between the answers provided to the open-ended questions and the classification results. Thus, we can observe that the 30.61% of conformists explain their decision in terms of a wish to conform to the FM (or to the FM and expectations of how the other SMs will behave). Examples of such reasons given:

*“I wanted to give the others some amount and I was willing to match the first mover up to $1.”*

*“I chose to transfer whatever the first mover decided on”*

Fairness/justice is another frequent explanation given by conformists (18.37%); fairness/justice is also frequently mentioned by compensators (31.25%), although their most frequently mentioned explanation is explicitly about compensation (37.5%). Examples of such statements include:

*“The more the first mover was to transfer, the less I was willing to transfer and vice versa. I didn't want the non-selectees to be left with nothing, but if they were already getting a good amount I didn't feel the need to transfer much to them out of my own pocket.”*

*“If they contributed a lot, then I wanted to contribute just a little bit. And if they contributed just a bit, then I contributed just a little more.”*

Fairness is the main reason provided by unconditional givers (53.66%), as expected. Examples of statements referring to fairness include:

*“The only fair thing to do was to transfer half of what I received to those who did not receive anything. I would do this no matter what I believed the first mover was going to do.”*

*“I thought .25 a fair amount to give, given the circumstances and what other people gave was of no significance to me.”*

And as we can observe, a great many (60.14%) self-interested SMs were quite content to explain their decision in very blunt terms, for example:

*“I simply decided that in order for me to gain the most out of the study, I should not transfer my money no matter what.”*

*“I'm selfish and want the most money.”*

Distribution of Open Ended Explanations Given by SM Type

Results also indicate that, overall, 26% of SMs listed as ‘other’ explain their decisions explicitly in terms of mixed strategies. Examples of these explanations include:

*“I mostly decided to simply match what the First Mover picked. The only exceptions is if it went beyond $0.25. I felt that was a fair amount.”*

*“I would match it up to 99c. if they chose a dollar, I would send nothing”*

Interestingly, 16% and 13% of conformists and compensators respectively explain their decisions in terms of mixed strategies.
