# Supplementary material for: The effect of anchors and social information on behaviour
Source: PLoS One. 2020 Apr 14;15(4):e0231203. doi: 10.1371/journal.pone.0231203 (PMC7156041; doi:10.1371/journal.pone.0231203)
Supplement: S1 Data — (DOCX) [file pone.0231203.s009.docx]

## EXPERIMENTAL INSTRUCTIONS

Note: a recording of the interactive platform can be found in the following link: [LINK REMOVED FOR REVIEW]

**INTRUCTIONS FOR ALLOCATORS**

Screen 1 (All)

Thank you for participating in this study. The aim of this research is to explore decision-making behavior. Please note that there are no “right” or “wrong” choices.

You will earn $0.75 for completing the survey. You will also have the chance of earning more money. The extra amount earned will depend on the choices that you and other participants make during the survey.

Please do not use the 'Back' and 'Forward' buttons in your browser.

We also kindly ask that you do not complete this survey on your i-phone due to compatibility issues with some of the question formats.

Please only participate in this study if you can commit about 10 minutes to it.

When you are ready, please move to the next page to start the survey.

Screen 2 (All)

*Please read the following instructions carefully. The rest of the survey depends on your comprehension of these instructions.*

For the purpose of this study, you will be randomly assigned to a group of 8 Mechanical Turk participants (including yourself) who will also be taking part in this study.

The computer will **randomly** select half of the participants within your group to receive a $2 bonus each.

The remaining participants in your group will **not** receive this extra $2.

If you are randomly selected to receive $2, you will have an opportunity to transfer some of this $2 to those who were not randomly selected for payment. You will then earn the portion of the $2 that you did not transfer (plus your $0.75 participation fee).

 If you are not randomly selected to receive the $2, then the total amount transferred by those who receive the bonus will be divided up equally among those of you who were not selected for payment.

In other words: if 4,8 of you (including yourself) are not randomly selected to receive the bonus, then you will each receive one quarter of the total amount transferred. You also, of course, receive the $0.75 for participation.

Please ensure that you have read and understood these instructions.

The following questions are designed to test whether you have read and understood the above scenario.

*You MUST answer the next two questions correctly to receive your bonus!*

Q1. If you have been randomly selected to receive the $2 bonus and you decide to transfer some of this to the participants who were not randomly selected for extra payment, how much will you receive at the end of this study (including the participation payment)?

| Amount | Check one only |
| --- | --- |
| $2.75 |  |
| $0.75 plus the amount I transferred to the other participants |  |
| $0.75 plus the amount left over from what I transferred to the other participants |  |
| $0.75 plus the amount the other participants transferred to me |  |
| $2 plus what I transferred to the other participants |  |

Q2. If you have been **not** been randomly selected to receive an extra $2, how much will you receive at the end of this study (including the participation payment)?

| Amount | Check one only |
| --- | --- |
| The total amount that the other participants have transferred |  |
| $0.75 plus an equal share of the total amount transferred |  |
| $2.75 |  |
| $2 |  |
| $0.75 |  |

Screen 3 (All)

Great!

As mentioned on the previous page, you will be randomly assigned to a group of 8 MTurk participants (including yourself) who will also be taking part in this study.

*You and all the participants in your group will be participating in this study simultaneously*.

This means that you may have to wait a few minutes until enough participants have accepted to complete this study and have been randomly assigned to the same group as you.

You will see the other participants at the top of the page as they join the group.

Once your group of 8 participants is complete, we will begin the study. This should only take a few minutes.

You will never know who the participants are in your group. The group composition is secret for every participant.

Screen 4 (All)

Your group of 8 is now complete! You are now ready to continue with this study.

The computer will **randomly** select half of the participants within your group to receive a $2 bonus each.

The remaining participants in your group will **not** receive this extra $2.

Any amounts that are transferred will be divided up between the participants who did not get randomly selected for payment. Should you require written confirmation that all transfers have been allocated correctly, please contact [STUDY AUTHOR].

Please note: you MUST complete the entire study to receive your participation payments and any earned bonus payments.

Please move to the next page to determine whether you have been selected to receive $2 or not.

Screen 5 (Allocators)

Remember that you have been randomly assigned to a group of 8 participants (including yourself).

In your group, **you and 3 other participants have been randomly selected to receive the $2 bonus.**

The remaining 4 participants in your group have **not** been selected to receive this bonus payment.

You will now have the chance to transfer some of the $2 to the participants who have not been selected for payment. The other participants who have randomly received $2 will also have the chance to transfer money.

However, before we proceed, the computer will now randomly select one of you to make a transfer *before anyone else*.

This person will be referred to as the ‘first-mover’. The transfer made by the first-mover *will be made visible to all the other participants*.

After the first-mover has made a decision, and this has been made visible to the rest of the group, all the remaining participants who received $2 may make their transfers. These transfers will not be made visible to other participants.

Please move to the next page to determine whether you have been selected to be the ‘first mover’.

Screen 6a (First-Movers)

**You have been randomly-selected selected to be the first-mover.**

You will now have the chance to transfer some of your $2 to the participants who have not been selected for payment. As the first-mover, you will make your decision before anyone else.

*All other participants in your group will see the amount that you transfer.*

After seeing this, those who also received $2 will have the chance to transfer some of their bonus to the 4 participants who received nothing.

*You may transfer as much or as little as you like.*

Q3. Please indicate how much of your $2 you would like to transfer to the other participants in your group who have not been randomly selected for payment:

*Select amount from below options:*

[$0] [$0.10] [$0.25] [$0.50] [$0.75] [$1]

Screen6b (Second-movers)

You have not been randomly-selected selected to be the first-mover.

Please wait a few moments whilst the randomly-selected first-mover makes their transfer. Once the first-mover has made a decision, *you will see how much they have chosen to transfer*.

Then you and all the other participants who have received $2 will have the chance to transfer some of your bonus to the 4 participants who have not been selected for payment.

The first-mover has been given the choice of transferring one of the following amounts:

[$0] [$0.10] [$0.25] [$0.50] [$0.75] [$1]

The selected amount will be distributed amongst the four participants who did not receive the $2 bonus, and shared equally among them.

Before seeing what the first-mover has transferred, please indicate what you will contribute in response to each of these possible first-mover transfers.

These transfer decisions are binding.

Once you have indicated how much you will transfer *given all possible first-mover transfers*, you will find out how much the first-mover has actually transferred. Then the corresponding amount that you indicated you would transfer in response to this first-mover transfer will be implemented.

*Remember: the decisions you make now are binding.*

*RANDOMLY VARY ORDER OF PRESENTATION:*

If the randomly-selected transfer is $0 I will transfer [*enter amount:……….*]

If the randomly-selected transfer is $0.10 I will transfer [*enter amount:……….*]

If the randomly-selected transfer is $0.25 I will transfer [*enter amount:……….*]

If the randomly-selected transfer is $0.50 I will transfer [*enter amount:……….*]

If the randomly-selected transfer is $0.75 I will transfer [*enter amount:……….*]

If the randomly-selected transfer is $1 I will transfer [*enter amount:……….*]

Please move to the next page to see how much the first-mover decided to transfer.

Screen 10a (Second-movers only)

The first-mover has decided to make a transfer of [AMOUT HERE] to the 4 participants who did not receive a bonus.

Screen 10b (Payment screen, second-movers only, if first-mover has dropped out of study)

The first-mover has decided to drop out of the study. There will be **no** first-mover in this study.

You will now have the chance to transfer some of the $2 to the participants who have not been selected for payment. The other participants who have randomly received a bonus will also have the chance to transfer money.

*You may transfer as much or as little as you like.*

*All transfers are confidential and cannot be traced back to you.*

Q4. Please indicate how much of your $2 you would like to transfer to the other participants in your group who have not been randomly selected for payment:

*Enter amount […..] (from $0 to $2)*

Screen 11 (Follow-up questions)

Now a few questions about your choice:

Q5. Can you explain in a few sentences how you decided upon the amount to transfer?

Screen 12 (All allocators)

Q6. How many of the other participants who were selected for payment in your group do you think made a transfer to the participants who received nothing?

ENTER NUMBER HERE: […………….]

Q7. Thinking only about those participants that you believe made a positive transfer: how much money do you think that each of them transferred on average?

ENTER AMOUNT HERE: $[…………....]

Screen 15 (Socioeconomic questions) (All players)

Now a few final questions about yourself.

Q14. Are you..?

*Tick one only*

[ ] female

[ ] male

[ ] other

Q15. In what year were you born?

…………

Q.16 What is the highest level of education you have received?

*Tick one only*

[ ] Less than high school

[ ] High school/ GED

[ ] 2-year college degree

[ ] 4-year college degree

[ ] Master’s degree

[ ] Doctoral degree

[ ] Professional degree (JD, MD)

Q17. What is your combined annual household income, before tax?

*Please remember that all answers are confidential. Income is a very useful measure for research purposes.*

*Check one only*

[ ] Less than $30,000

[ ] $30,000-$39,999

[ ] $40,000-$49,999

[ ] $50,000-$59,999

[ ] $60,000-$69,999

[ ] $70,000-$79,999

[ ] $80,000-$89,999

[ ] $90,000-$99,999

[ ] $100,000-$124,999

[ ] $125,000-$149,999

[ ] $150,000 or more

Q18. How many children under 16 years old live in your household?

*Check one only*

[ ] 0

[ ] 1

[ ] 2

[ ] 3

[ ] 4 or more

Q19. Do you consider yourself to be a Democrat, Republican, Independent or Other?

*Check one only*

[ ] Democrat

[ ] Republican

[ ] Independent

[ ] Other

Screen 17 (World Values Survey Trust Q)

Q20. Generally speaking, would you say that most people can be trusted or that you need to be very careful in dealing with people? (select one answer):

[ ] Most people can be trusted.

[ ] Need to be very careful.

Screen 18 (End of Survey)

Thank you for your time!

You have earned $0.75. Any extra earning will be credited into your account when the study is complete.

**If you would like to add any further comments please use the space below:**

|  |
| --- |

**INTRUCTIONS FOR RECIPIENTS**

--Screen 1 to 4 as for allocators--

Screen 5R (Recipients)

In your group, you and other participants (shown above) have not been randomly selected to receive the $2 bonus.

The remaining participants in your group have been selected to receive this bonus payment.

The participants that have been randomly selected to receive a bonus of $2 each will now have the chance to transfer some of the $2 to those of you who were not selected to receive the bonus. They may transfer as much or as little as they like.

*Go to Screen 15*

--Screen 15 to 18 as for allocators--
