# Supplementary material for: The effect of anchors and social information on behaviour
Source: PLoS One. 2020 Apr 14;15(4):e0231203. doi: 10.1371/journal.pone.0231203 (PMC7156041; doi:10.1371/journal.pone.0231203)
Supplement: Attachment — Submitted filename: Response to reviewers R2.docx [file pone.0231203.s011.docx]

PONE-D-19-24674R1
The Effect of Anchors and Social Information on Behaviour
PLOS ONE

Dear PhD O'Garra,

Thank you for submitting your revision to PLOS ONE, as you will see from the referee reports, the paper has been substantially improved in their opinion and I share this judgment. However, there is still some issues, related mostly to presentation and clarity of your writing. Please, take the last effort to perfect your paper (hence: minor revision). Please, mark clearly your improvements relative to the current version of the text upon submission. If possible, please, have the text proof-read by a professional writer, to facilitate the flow and make your article better received by the audience.

We would appreciate receiving your revised manuscript by Feb 22 2020 11:59PM. When you are ready to submit your revision, log on to https://www.editorialmanager.com/pone/ and select the 'Submissions Needing Revision' folder to locate your manuscript file.

If you would like to make changes to your financial disclosure, please include your updated statement in your cover letter.

To enhance the reproducibility of your results, we recommend that if applicable you deposit your laboratory protocols in protocols.io, where a protocol can be assigned its own identifier (DOI) such that it can be cited independently in the future. For instructions see: [http://journals.plos.org/plosone/s/submission-guidelines#loc-laboratory-protocols](https://eur02.safelinks.protection.outlook.com/?url=http%3A%2F%2Fjournals.plos.org%2Fplosone%2Fs%2Fsubmission-guidelines%23loc-laboratory-protocols&data=02%7C01%7Ct.ogarra%40mdx.ac.uk%7Cd48eafb4fcba41138e1d08d7942ce85f%7C38e37b88a3a148cf9f056537427fed24%7C0%7C0%7C637140792903387162&sdata=AsBGRqjGQi9%2BtamtQCdx2H7w6MlMPY26SZax5OvltAQ%3D&reserved=0)

Please include the following items when submitting your revised manuscript:

- A rebuttal letter that responds to each point raised by the academic editor and reviewer(s). This letter should be uploaded as separate file and labeled 'Response to Reviewers'.
- A marked-up copy of your manuscript that highlights changes made to the original version. This file should be uploaded as separate file and labeled 'Revised Manuscript with Track Changes'.
- An unmarked version of your revised paper without tracked changes. This file should be uploaded as separate file and labeled 'Manuscript'.

Please note while forming your response, if your article is accepted, you may have the opportunity to make the peer review history publicly available. The record will include editor decision letters (with reviews) and your responses to reviewer comments. If eligible, we will contact you to opt in or out.

We look forward to receiving your revised manuscript.

Kind regards,
Joanna Tyrowicz
Academic Editor
PLOS ONE

Dear Prof Tyrowicz,

Many thanks. We have endeavoured to address the suggested revisions to the best of our ability. Any page references are made in relation to the unmarked Manuscript.

Regards,

Tanya O’Garra

Reviewers' comments:

6. Review Comments to the Author

Reviewer #1: To me, the paper has been substantially improved upon this revision. I have only minor comments/questions. Other than that, I think the paper is good for publication in the journal.

The authors could make sure that details regarding their study, in particular, in the description of the experiment, are provided sequentially in such an order that is understood to a reader unfamiliar with the study. I mean that a reader learns paragraph-by-paragraph about the study and does not need to wonder what some information means before reaching further parts of the paper. Examples:
(i) p. 7, “… hence drop-outs were not observed by SMs …” – this would not be clear to me as a reader without learning about the experiment structure in next sections of the experiment description. Hence, maybe it would be better if the subsection “Participants” was placed at the end of the section describing methods.

As suggested, the ’Participants’ section has been moved to the end of the methods section. Given this change of location, some of the text in this section has been adjusted or moved within the section to ensure that the text flows correctly, hence improving clarity of reading. In addition, the introductory sentences of the ‘Experimental Design’ section have also been adjusted due to the change in location, to improve readability and flow.

(ii) p. 14, the paragraph below the equations says about the analysis focused only on self-interested strategy. At the stage there, it is not clear why it is done so, and it sounds confusing.

To make this clearer, we have adjusted the text on page 14 to read:

“To explore whether the adoption of different strategies is affected by the initial information or ‘anchor’, we conduct a multinomial logistic regression on the different player ‘types’, as well as a binary logistic regression specifically aimed at addressing whether anchors the adoption of a ‘self-interested’ strategy. Our motivation for focusing on the ‘self-interested’ type is based on our finding that this particular behavioural strategy appears to be most susceptible to anchors.”

We have also removed the sentence saying that the multinomial regression has been placed in the Online Appendix, and instead make this point – and the justification for doing so – in the Results section. This makes for more clarity in reading the Analysis Procedures section.

The paragraph on p. 11 describes potential explanations. I know that it is a side discussion for this paper, nevertheless, the paragraph sounds a little misleading to me. It suggests as if there are only three possible explanations, although I think there could be many more, and these other explanations are provided in the literature too. For example, learning, fatigue or willingness to behave consistently. I do not mean to largely extend this side discussion in the paper but just to be careful in order not to suggest fewer explanations than the literature actually provides.

The proposed psychological mechanisms presented on page 6-7 are the main ones used to explain anchoring in the literature (reviewed in Cho, 2011), which is why we refer to these. However, we have acknowledged the possibility of other explanations as follows on page 7:

“However, we do not propose to identify whether these (or indeed, other explanations) explain our findings.”

Getting to known the screen as displayed to second-movers (Figure 1), it seems to me that the paper could include a short note on advanced disclosure. There was a reason for which the authors decided to present upfront all possible first-movers’ amounts to second-movers, and this could be explained. Especially that in some literature, advanced disclosure has been suggested to mitigate order/anchoring effects – e.g., Day, B., Bateman, I. J., Carson, R. T., Dupont, D., Louviere, J. J., Morimoto, S., Scarpa, R., and Wang, P. (2012). Ordering effects and choice set awareness in repeat-response stated preference studies. Journal of Environmental Economics and Management, 63(1), 73-91.

We have added the following paragraph to the Experimental Design section (page 11) clarifying our use of “advanced disclosure” of FM transfers to SMs:

“As a side note, we mention that the strategy method is usually used non-sequentially, i.e. subjects view all possible choices by another subject/other subjects and provide their conditional choices simultaneously. Thus, in the standard approach, subjects make their choices under a scenario of “advanced disclosure”. Given our interest in identifying whether subjects would anchor their decisions to the first amount they were presented with, we used a sequential approach. However, to keep our design as close as possible to the standard approach, we opted for advanced disclosure of the FM’s choices. Only when choices were to be made, was this done sequentially.”

The discussion on p. 26 talks about the analysis of expectations. I do not know whether it is my mistake but I have missed information in the paper how the data on expectations was collected (e.g., what type of questions and in which part of the experiment). As this is used for the discussion in the paper, I think it would be good to inform a reader about these few details.

This has been clarified in the Experimental Design section (page 11) as follows:

“Participants then indicated how much they expected other SMs in their group to contribute on average.”

Given the equation on p. 14, I think the text in Table 3 could be adjusted. Specifically, the text informs about “beta”, while the equation includes two betas.

Thank you for noting this. Table 3 has been adjusted to reflect the two different betas used.

In Table 4, the word “selfish” could probably be changed for “self-interested” for consistency. This has been fixed

The text refers to section numbers, although the numbers are not present in the paper.

These have been removed, and/or section titles referred to instead of section numbers.

There are some punctuation and other mistakes that need corrections. Examples:
- p. 10, “(other examples include [40].” – missing end bracket fixed
- p. 11, “[41,42]and” – missing space fixed
- p. 13, “a Kruskal-Wallis (KW) test, which a rank-based nonparametric test” – missing “is” fixed
- p. 17, “transfers. in the models” – a large letter needed fixed
- p. 24, “(with $0,50 as the reference)” – a dot instead of a comma fixed
- inconsistent use of the word “dropouts” either with a dash or without it thanks for noting this – we have chosen to go with “dropout” and have corrected other spellings

Reviewer #2: This version has been highly improved; the richness of the data is presented clearly. I have only several comments.
1. On flow of writing: It might be helpful if the author mentioned clearly in the beginning that this study (particularly for RQ 2) is an exploratory and therefore readers could expect many interesting findings from this study following their hypotheses. As for the RQ 1, I suppose that actually the authors proposed an explicit hypothesis but it was written a bit far behind (p. 11 par 1), and some additional hypotheses about the anchor effect on female and age in the previous part (p. 10, par 1). I wonder if the “Identifying Anchor Effect” part (p. 10) can be moved up to right after Introduction. This is to ensure the link between literatures, research questions and hypothesis proposed by the author kept closed and therefore easily understood by the reader.

On page 4 (end of para 1) we have added the following text clarifying that this is exploratory research:

“This is exploratory research, and as such, we have no expectations about the size or direction of anchoring effects on the distribution of ‘types’ in the population under study. Our aim is mainly to identify whether the choice of behavioural strategy is affected by normatively irrelevant contextual factors, such as anchors.”

We also moved the section ‘Identifying Anchoring Effects’ so that is now right after the Introduction, as suggested. Given this change of location, some of the text in this section has been adjusted or moved within the section to ensure flow and readability. Table 1 and the accompanying text (which were in the ‘Identifying Anchoring Effects’ section) have now been moved to the ‘Experimental Design’ section, with some minor adjustments to the accompanying text. This has been done to maintain readability, given the change of location of various sections in the paper.

2. I understand that the authors have no intention to go further to explain the cognitive mechanism behind redistributive behaviour in this setting. But, since it is also written in the beginning that the focus of this study is redistributive behaviour, rather than cooperative behaviour (p.3 par 2) it may also important to relate this argument here with the argument of explanation used in the Result and Discussion part, that is: the influence of social information toward redistribution and behavioural strategy.

We have acknowledged that anchors may affect other pro-social behaviours, by adding a sentence in the Discussion and Conclusions (page 29) proposing that future research might address how anchors affect other pro-social behaviours, such as cooperation.

3. On reporting data and discussion. The authors may consider to add explanation of the result based on the effect size. The effect size will be important to be used when discussion the result and what are suggested further research, regardless the p-value. I really appreciate the qualitative data on types of behavioural strategy. I agree this is very important findings. Just one little questions regarding the open ended data processing (whether you are using interrater or not, etc.). This is perhaps can be added, just to be sure.

Effect sizes with respect to results in Table 2 have now been reported on page 21, and effect sizes are now mentioned in the Discussion and Conclusions section (page28), in addition to the statistical significance, as suggested.

As for the open-ended data, it is explained in the Online Appendix S8 that the data was coded by three people and final codes agreed on through discussion. We have also added a measure of interrater reliability (Gwet’s AC, value of 0.56) to the Online Appendix.
