# Supplementary material for: The effect of anchors and social information on behaviour
Source: PLoS One. 2020 Apr 14;15(4):e0231203. doi: 10.1371/journal.pone.0231203 (PMC7156041; doi:10.1371/journal.pone.0231203)
Supplement: Attachment — Submitted filename: Response to Editor regarding R3.docx [file pone.0231203.s012.docx]

PONE-D-19-24674R2
The Effect of Anchors and Social Information on Behaviour
PLOS ONE

Dear PhD O'Garra,

thank you for submitting the revised version of your study. I think both referees would be fully satisfied. I read your paper carefully and there are two issues which I find a bit problematic. Feel free to answer me directly by emailing to j.tyrowicz@uw.edu.pl if you find these questions simply wrong. However, if I am right, I would expect you to fix those minor issues, upon which your paper can be accepted at PLOS ONE.

Dear Prof Tyrowicz,

Thank you for your suggestions – please see below for my responses and amendments. I hope these are to your satisfaction.

Regards,

Tanya

Issue #1. When I look at Table 2, it is clear to me what is the reference level for your dummies in Column 1, but in Column 3 you interact your IA dummies with the amount. In principle it can be informative, but I am confused about the base levels in this case. As you have 0 for IA!=1 and a given amount, it seems to me that the base level in Column 3 is both 0 for each level of IA and on top of that IA=0.5$. I find that confusing and given the interaction term, you can use all the levels in Column 3.

Following our email conversation, I have added a sentence to the table clarifying that the reference level for the IA is $0.50 (this text is also included in the main text).

Issue #2. Table 2 reports a mixed effects regression, but nowhere in your paper do I find the assumption concerning the standard error, i.e. I think you should cluster standard errors at SM level, because presumably all the responses of a given SM are driven by the same decision rule (i.e. they are not independent). If Table 2 reports clustered standard errors, I think it should be made salient. If they are not clustered, I fear that you may need to re-estimate the model and report the new standard errors in your Table 2.

These are minor issues. If re-estimating your model necessitates other changes in text, please do so. As minor minor points, I can bring to your attention the following:

As suggested, I have re-run the models with clustering of standard errors (using vce(cluster id) as suggested). This has resulted in some changes to the regression results which have now been updated in the manuscript. As a result, I have had to rewrite some of the text to account for these changes. For consistency, I have also re-run the extra models reported in Online Appendix S5 also with clustering of standard errors at the individual (MM) level.

* I think it is typical to use present simple tense in reporting the findings of the literature and your own (rather than present continuous, or other continuous).

Thanks for noting this, it has been corrected.

* Perhaps your paper is a bit long both in words and in the illustrations. I leave it at your discretion for the text. As to the illustrations, personally, I see little value to Figure 1 and 2, they can very well be reported in one Table (if need be) and it may confuse some readers not to see the CI whiskers (even despite your notes).

Note: we clarified by email that you were referring actually to Figs 3 and 4.

To shorten the length of the paper, I have put Figure 3 in the Online Appendix S2 together with the distributions of SM responses to FM transfers. I have not included standard error bars as this is not correct with repeated measures data (as clarified in Estes, 1997) and box plots are not suitable for continuous two-way data. However, by placing the line graph together with the histograms showing the distributions of responses to each FM transfer, it should be clear to the reader that these averages come from distributions of responses. To further emphasise the link between the distributions and the line graph, I have added the following sentence to the Online Appendix:

“In the following figure we summarise the above distributions in the form of a line graph depicting mean SM transfers in response to each FM transfer.”

Please note that the above comments have no influence on whether or not your paper is accepted for publication in PLOS ONE. The decision is positive and will not change if your results change (e.g. because of clustering). These are my requests made in the interest of presenting the highest quality research to our audience. Note also that if I am mistaken in the two issues numbered above, I am happy to hear your comments. Maybe they help us clarify the text so that other readers would not be confused.

We would appreciate receiving your revised manuscript by Mar 27 2020 11:59PM. When you are ready to submit your revision, log on to https://www.editorialmanager.com/pone/ and select the 'Submissions Needing Revision' folder to locate your manuscript file.

If you would like to make changes to your financial disclosure, please include your updated statement in your cover letter.

To enhance the reproducibility of your results, we recommend that if applicable you deposit your laboratory protocols in protocols.io, where a protocol can be assigned its own identifier (DOI) such that it can be cited independently in the future. For instructions see: [http://journals.plos.org/plosone/s/submission-guidelines#loc-laboratory-protocols](https://eur02.safelinks.protection.outlook.com/?url=http%3A%2F%2Fjournals.plos.org%2Fplosone%2Fs%2Fsubmission-guidelines%23loc-laboratory-protocols&data=02%7C01%7Ct.ogarra%40mdx.ac.uk%7C4be0f5d5ec424488927608d7aeedbe01%7C38e37b88a3a148cf9f056537427fed24%7C0%7C0%7C637170208426372227&sdata=yQGul2IVTfQkqC0TGnoxYnVVYFl2OgyIWbKynZ0%2BGQ8%3D&reserved=0)

Please include the following items when submitting your revised manuscript:

- A letter that responds to my points raised above. This letter should be uploaded as separate file and labeled 'Response to Reviewers'.
- A marked-up copy of your manuscript that highlights changes made to the original version. This file should be uploaded as separate file and labeled 'Revised Manuscript with Track Changes'.
- An unmarked version of your revised paper without tracked changes. This file should be uploaded as separate file and labeled 'Manuscript'.

Please note while forming your response, that you may have the opportunity to make the peer review history publicly available. The record will include editor decision letters (with reviews) and your responses to reviewer comments. If eligible, we will contact you to opt in or out.

We look forward to receiving your revised manuscript.

Kind regards,

Joanna Tyrowicz
Academic Editor
PLOS ONE


[NOTE: If reviewer comments were submitted as an attachment file, they will be attached to this email and accessible via the submission site. Please log into your account, locate the manuscript record, and check for the action link "View Attachments". If this link does not appear, there are no attachment files to be viewed.]


While revising your submission, please upload your figure files to the Preflight Analysis and Conversion Engine (PACE) digital diagnostic tool, [https://pacev2.apexcovantage.com/](https://eur02.safelinks.protection.outlook.com/?url=https%3A%2F%2Fpacev2.apexcovantage.com%2F&data=02%7C01%7Ct.ogarra%40mdx.ac.uk%7C4be0f5d5ec424488927608d7aeedbe01%7C38e37b88a3a148cf9f056537427fed24%7C0%7C0%7C637170208426382228&sdata=e13dFzXsOv6umC8PxHgYNy%2FdKSJW1yUsboYu0SLAqaE%3D&reserved=0). PACE helps ensure that figures meet PLOS requirements. To use PACE, you must first register as a user. Registration is free. Then, login and navigate to the UPLOAD tab, where you will find detailed instructions on how to use the tool. If you encounter any issues or have any questions when using PACE, please email us at [figures@plos.org](mailto:figures@plos.org). Please note that Supporting Information files do not need this step.

*In compliance with data protection regulations, you may request that we remove your personal registration details at any time.* [*(Remove my information/details)*](https://eur02.safelinks.protection.outlook.com/?url=https%3A%2F%2Fwww.editorialmanager.com%2Fpone%2Flogin.asp%3Fa%3Dr&data=02%7C01%7Ct.ogarra%40mdx.ac.uk%7C4be0f5d5ec424488927608d7aeedbe01%7C38e37b88a3a148cf9f056537427fed24%7C0%7C0%7C637170208426392222&sdata=eDNXA48J%2BAssQj%2FoGssKXYbYMn%2F6r5l8GJC3S2ERl9o%3D&reserved=0)*. Please contact the publication office if you have any questions.*
